# Menopausal Age and Chronic Diseases in Elderly Women: A Cross-Sectional Study in Northeast China

**DOI:** 10.3390/ijerph13100936

**Published:** 2016-09-22

**Authors:** Yingli Fu, Yaqin Yu, Shibin Wang, Joseph Sam Kanu, Yueyue You, Yingyu Liu, Yangyu Zhang, Yawen Liu, Bo Li, Yuchun Tao, Changgui Kou

**Affiliations:** Department of Epidemiology and Biostatistics, School of Public Health, Jilin University, Changchun 130021, Jilin, China; fuyingli318@126.com (Y.F.); yuyaqin5540@163.com (Y.Y.); spiriorwang@126.com (S.W.); samjokanu@yahoo.com (J.S.K.); yyy841109@163.com (Y.Y.); liuyy15@mails.jlu.edu.cn (Y.L.); yyzhang15@mails.jlu.edu.cn (Y.Z.); ywliu@jlu.edu.cn (Y.L.); libo2016tougao@sina.com (B.L.); taoyuchun@163.com (Y.T.)

**Keywords:** menopausal age, chronic disease, elderly women

## Abstract

Many factors affect menopausal age, and early or late onset of menopause may be associated with many chronic health problems. However, limited data are available regarding this phenomenon in the Northeast China population. For this study, 2011 elderly women were selected as a sample from participants in a cross-sectional survey performed using stratified, clustered multistage, and random sampling methods. Early menopause was more prevalent in subjects born from 1943 to 1947 (OR = 1.708, 95% CI = 1.205, 2.420) and 1933 to 1937 (OR = 2.445, 95% CI: 1.525, 3.921) and in physical laborers (OR = 1.413, 95% CI = 1.021, 1.957). Women with less than nine years of education (OR = 0.515, 95% CI: 0.327, 0.812) and who were current smokers (OR = 0.577, 95% CI: 0.347, 0.959) were less likely to have late menopause. BMIs between 25 and 30 (OR = 1.565, 95% CI: 1.152, 2.125) and greater than 30 (OR = 2.440, 95% CI: 1.482, 4.016) were associated with later menopausal age. Late menopause was positively associated with diabetes (OR = 1.611, 95% CI: 1.142, 2.274) but protective against chronic gastroenteritis/peptic ulcers (OR = 0.533, 95% CI: 0.333, 0.855). Results showed that (1) Being born in an earlier year, having a lower education, and engaging in physical labor were associated with an earlier menopausal age, while a higher BMI was associated with a later menopausal age; and that (2) menopausal age was associated with diabetes and gastroenteritis in elderly women living in Northeast China.

## 1. Introduction

Menopause is defined as the absence of menstruation periods for at least twelve months in the absence of pregnancy. Women are defined as having early menopause if they experience menopause before 45 years old [1]. Due to exposure to different hormonal environments, early or late onset of menopause may be associated with many chronic health problems. Early menopause has been reported to be associated with all-cause mortality [2,3] and has numerous adverse effects on cardiovascular disease [4], neurological diseases, and osteoporosis [5]. However, most evidence on the link between menopausal age and health conditions is from Western populations, with only a few studies previously conducted in Asian populations. Most previous studies have focused on physical diseases, such as cardiovascular disease [6], parkinsonism [6], heart failure [7], and stroke [8], and no studies have focused on mental disorders. Little is known about the factors that affect the timing of menopause, although several lifestyle-related factors have been suggested [9]. Early menopause has been associated with smoking [10], low body mass index (BMI) [11], and less education [12], while late menopause has been associated with marriage and cohabitation [13]. It is unclear which factors are associated with menopausal age among women from Northeast China. Given that the lifestyle of people in Northeast China (high salt and fat content diets, and extremely low temperatures during winter) is different from people in other parts of China, our findings will be meaningful and enable us to provide a theoretical basis for the management of menopause-associated problems when the government makes relevant health policies. The main objective of this study was to analyze the effects on menopausal age and explore the associations between menopausal age and twelve common chronic diseases, including mental health, in elderly women from Northeast China.

## 2. Materials and Methods

This study was part of a Chronic Disease Survey conducted from June 2012 to August 2012 in Jilin, a province in Northeast China with approximately 27.5 million inhabitants.

### 2.1. Sampling

Altogether, 21,435 respondents participated in this survey, giving a response rate of 84.9%. For this study, we included 2011 elderly women aged 60 to 79 years who had lived in the Jilin Province for more than 6 months. People aged ≥60 years were classified as “elderly persons” in China. This age cut-off has been used in other studies [14,15,16]. This sample can be considered as representative of the whole population of elderly women in the Jilin Province because participants were recruited using a multistage stratified cluster sampling method. More details regarding the sampling procedure are described elsewhere [17].

### 2.2. Data Collection and Measures

All participants were interviewed face-to-face at local community clinics or health service centres by trained interviewers. Data on basic sociodemography, mental health, chronic disease, and related lifestyle factors were collected. In addition, we measured participant blood pressure, fasting plasma glucose (FPG), fasting total cholesterol (TC), triglycerides, high-density lipoprotein (HDL), and low-density lipoprotein (LDL) levels. The assessment protocols are described below:

#### 2.2.1. Life-Style Factors

(1) Current smoking was defined as smoking at least 1 cigarette every day in the past 30 days; (2) Current moderate or heavy drinking was defined as consuming on average more than 3 alcoholic drinks per week [18]; (3) Dietary habits were dichotomized as poor and regular eating habits; (4) Lack of exercise referred to exercising less than three times per week, which was defined as active and conscious physical activity; (5) BMI was calculated as weight/height^2^ (kg/m^2^). Adults were categorized as “normal or underweight” (BMI < 25 kg/m^2^), “overweight” (25 ≤ BMI < 30 kg/m^2^), or “obese” (BMI ≥ 30 kg/m^2^) according to the international classification of the World Health Organization (WHO) [19]; (6) Personal monthly income took into account the total family income and the number of family members in a household, and was classified into three groups in line with the classification of the Jilin Provincial Bureau of Statistics: low (<1000 yuan/month), middle (1000–3000 yuan/month), and high income (>3000 yuan/month); (7) Physical laborers were defined as production workers, farmers, and service workers; otherwise, “unclassified laborers” were defined as officers, technical staff, students, the unemployed, and retirees.

#### 2.2.2. Menopausal Age

Menopause was defined as the absence of menstruation periods for at least twelve months in the absence of pregnancy [1]. Women were asked if they had experienced “menopause”, and the age at which they experienced menopause was inquired. Participants were defined as having early menopause if they experienced menopause before 45 years old [1]. Late menopause was defined as experiencing menopause after 54 years old. Women who experienced menopause between 46 and 53 years old were defined as having experienced normal menopause.

#### 2.2.3. Poor Mental Health

The Chinese version of the 12-item General Health Questionnaire (GHQ-12) was used to evaluate the general mental health status of the participants during the previous month [20]. All participants were divided into two groups based on GHQ-12 total scores, which range from 0 to 12, with a score of 4 as the cut-off point. Participants with scores ≥4 were classified as having poor mental health [21,22].

#### 2.2.4. Chronic Diseases

Chronic diseases were listed according to the International Classification of Disease, 10th Revision (ICD-10) and included anemia, diabetes, hyperlipidemia, hypertension, ischemic heart disease, cerebrovascular diseases, chronic obstructive pulmonary disease (COPD), chronic gastroenteritis/peptic ulcers (CGPU), chronic cholecystitis/gallstones, arthritis, and chronic lower back pain. These chronic diseases were diagnosed by general practitioners through evaluation of physical examination and laboratory results. Diabetes was defined according to the WHO criteria as FPG ≥ 7.0 mmol/L (126 mg/dL), a self-reported previous diagnosis established by general practitioners, or both. Hyperlipidemia was defined as having at least one of the following: high total cholesterol (TC ≥ 6.22 mmol/L), high triglycerides (TG ≥ 2.26 mmol/L), low high-density lipoprotein (HDL < 1.04 mmol/L), high low-density lipoprotein (LDL ≥ 4.14 mmol/L), and having a history of dyslipidemia in the past years. Other chronic diseases were judged only by self-reported history of diseases diagnosed by general practitioners. “Any disease” was defined as one or more of the 11 chronic diseases or poor mental health.

### 2.3. Data Analysis

Analyses were carried out using the SPSS Complex Samples version 21.0 package (IBM SPSS, IBM Corp, Armonk, NY, USA). Data were weighted by gender, age, administrative regions, and place of residence (urban vs. rural area) according to census data from the Jilin Provincial Bureau of Statistics. Descriptive statistics for all variables were presented according to menopausal age and analyzed using Rao-Scott χ^2^ tests for comparison of proportions based on the complex sampling design. Two analyses were conducted. Firstly, the effects of sociodemography and lifestyle factors on menopausal age were analyzed using multivariate logistic regression analysis (cslogistic command in SPSS) with menopausal age as the dependent variable and marital status, living area, income, education, and lifestyle factors as the independent variables. Secondly, independent associations between specific chronic diseases and menopausal age were explored using multivariate logistic regression models. Each chronic disease was used as a dependent variable, while menopausal age was used as the independent variable, and demographic and lifestyle factors were entered as confounding factors into the multivariate logistic regression analyses. Multi-collinearity between independent variables was detected using a variance inflation factor and correlation matrix. Results were considered significant when *p* < 0.05 (two-sided).

### 2.4. Ethics

The study protocol was approved by the Health Bureau of Jilin Province (Reference number: 2012-10) and the Ethics Committee of the School of Public Health, Jilin University (Reference number: 2012-R-011). All participants were informed of the procedures of this research study and signed consent forms prior to taking part in the survey.

## 3. Results

### 3.1. Basic Subject Information

Demographic and lifestyle characteristics of the study subjects are presented according to menopausal age in Table 1. We found that menopausal age was significantly different among women with different birth years (*p* = 0.008), and menopausal age also differed by educational level (*p* = 0.015), smoking status (*p* = 0.009), and BMI (*p* = 0.003). We found no significant differences in menopausal age by marital status, living environment, income, labor type, drinking, diet, or exercise status (all *p* > 0.05).

### 3.2. Chronic Diseases According to Menopausal Age

Table 2 presents the frequency of chronic diseases in relation to the various menopausal age groups. The frequency of diabetes and poor mental health were significantly different among different menopausal age groups. The frequencies of diabetes in women with early menopause, normal menopause, and late menopause were 17.0%, 65.2%, and 17.7%, respectively (*p* = 0.007). The frequencies of poor mental health in women with early menopause, normal menopause, and late menopause were 19.5%, 69.7%, and 10.8%, respectively (*p* = 0.033).

### 3.3. Effects of Different Sociodemographic and Lifestyle Factors on Menopausal Age

Table 3 shows the results of the multivariate logistic regression analyses of the effects of sociodemographic and lifestyle factors on menopausal age. Compared with the reference group (born from 1948 to 1952), early menopause was more prevalent in subjects born from 1943 to 1947 (Odds ratios (OR) = 1.708, 95% confidence interval (CI) = 1.205, 2.420) and 1933 to 1937 (OR = 2.445, 95% CI: 1.525, 3.921). As regards the type of work, early menopause was more prevalent in physical laborers than unclassified laborers (OR = 1.413, 95% CI = 1.021, 1.957). Women who received less than nine years of education (OR = 0.515, 95% CI: 0.327, 0.812) and were current smokers (OR = 0.577, 95% CI: 0.347, 0.959) were less likely to have late menopause. Additionally, there was a correlation between BMI and menopausal age. BMIs between 25 and 30 (OR = 1.565, 95% CI: 1.152, 2.125) and greater than 30 (OR = 2.440, 95% CI: 1.482, 4.016) were significantly associated with later menopausal age.

### 3.4. Effects of Menopausal Age on Chronic Diseases and Mental Health

Table 4 presents the results of the multivariate logistics regression analyses of effects of menopausal age on the 12 chronic diseases investigated and mental health. After an adjustment for potential confounding factors, we found that late menopause was significantly and positively associated with diabetes (OR = 1.611, 95% CI: 1.142, 2.274) but a protective factor against CGPU (OR = 0.533, 95% CI: 0.333, 0.855).

## 4. Discussion

The aim of this study was to identify factors that affect the onset of menopause in elderly women living in Northeast China and to analyze the effect of menopausal age on chronic disease prevalence.

The timing of menopause is an indicator of ovarian function and ageing and, therefore, critical for women’s health. Both early and late menopause have been shown to be associated with adverse health outcomes, highlighting the importance of the identification of factors across the life course that can affect menopausal age [23]. According to our results, there is evidence to suggest that birth year, education, type of work, current smoking status, and BMI are associated with menopausal age. While previous studies have investigated the effects of demography or lifestyle factors on menopausal age, their results have been inconsistent and inconclusive.

According to Ramezani Tehrani et al., the menopausal age among Tehrani women born in the 1930s, 1940s, and 1950s indicated a secular increasing trend [24]. Pakarinen et al. found that the menopausal age of women in Finland increased by one year during 1997–2007 [25]. In a survey of over 1400 Swedish women born between 1908 and 1930, Rodstrom and colleagues found a progressive and significant increase in the mean age at natural menopause [26]. Our results were consistent with these previous studies. In our study, women who were born in 1933–1937 had more than twice the risk of earlier menopausal age than women born in 1948–1952, and those who were born between 1943 and 1947 had 1.7 times greater risk of earlier menopause compared to the reference group. The 95% CI for the OR for birth in 1938–1942 compared to the reference group (0.922–1.946) nearly exceeded the null hypothesis value. Therefore, we could regard it as being marginally associated with an earlier onset of menopause. According to previous studies, menopausal age has a secular increasing trend; within our data, a relative but not a linear trend was indicated. Women who were born in 1933–1947 experienced a long period of war (from 1937 to 1949) during their childhood and a serious famine (from 1959 to 1961) during puberty. Lack of nutrition and psychological pressure may be correlated with their physiological status. However, not all studies support this conclusion. In a review of population-based studies, Mckinlay et al. in 1996 reported that there was no evidence of any secular trend in menopausal age [27].

Results of a longitudinal study of 5961 Australian female twins reported that higher education was associated with later menopausal age [28]. We obtained similar results in this study, finding that women living in Northeast China who received more than nine years of education had a higher menopausal age. A study conducted in a Lebanese population showed that education was not significantly associated with the menopausal age [29]. Birth year may be a confounding factor in the relationship between educational level and menopausal age because most women born before 1960 in China have significantly lower levels of education, and our study subjects were all born before 1952. However, after adjusting for birth year, our results remained significant.

In a study by Magursky and colleagues, the researchers reported that women working in agriculture and as housewives had a slightly later mean menopausal age, whereas manual workers had menopause approximately one year earlier [30]. However, our results showed that physical laborers had earlier menopause than unclassified workers. The mechanism underlying this association remains unclear but may be at least partially explained by lifestyle factors such as smoking and diet. Although most studies have adjusted for lifestyle factors such as smoking and BMI, no adjustments have previously been made for dietary factors [23]. Occupation may be a factor correlated with educational level, as people with lower education tend to be manual workers. Additionally, both education and occupation have been found to be determinants of household income; therefore, general nutritional status, standard of living and access to healthcare services may contribute to the relationship between type of work and menopausal age [31].

Many previous studies, including a meta-analysis, have confirmed that exposure to cigarette smoke is a risk factor for early menopause [23,32,33]. This conclusion was further supported by our study. In terms of biological mechanisms, smoking has been associated with altered hormone production and metabolism, including expression of the *CYP1A2* genotype, decreased serum estrogen levels [34], and increased 2-hydroxyestrogen concentration [35] and androgen quantity [36]; all of these factors may contribute to an anti-estrogenic effect that results in earlier natural menopause.

Previous studies have reported that increased BMI was associated with later menopause onset [26,37]. Our results showed that menopausal age for women whose BMI was >25 was later than those who had a BMI <25. Overweight and obese women have higher circulating levels of estrogen coupled with lower levels of sex hormone-binding globulin, and these conditions may result in delayed menopause [38]. However, several studies have shown that the extremes of BMI were associated with menopausal age, with both higher [39] and lower [11] BMI values identified as being associated with earlier menopause. Furthermore, some studies have found no association between BMI and menopausal age [25,40]. These inconsistent results may be due to study populations that differed in terms of ethnicity and culture and the use of different analytical methods, and varying results across studies could also be due to variations in the samples or covariates included in models. In addition, cigarette smoking has been identified as a strong confounding factor due to its relationship with both a lower BMI and early menopause [41].

Menopausal age may be associated with many diseases, such as diabetes, gastroenteritis, and hypertension. The results of our study also suggest that diabetes and poor mental health are correlated with menopausal age. However, after controlling for confounders, only diabetes and CGPU were significantly associated with menopausal age. The association between diabetes and early menopause has been examined previously; however, results have been inconsistent. Studies conducted in both South China and Japan suggested that no association existed between menopausal age and diabetes [42,43]. The results from the European Prospective Investigation showed that women with an onset of diabetes before the age of 20 years reached menopause earlier, whereas onset of menopause was delayed in women having diabetes after 50 years of age [44]. Another study by Monterrosa-Castro and colleagues also found diabetes to be associated with early menopause in women under the age of 45 years [45]. There is some biological evidence that anti-Mullerian hormone (AMH) levels can be used to predict menopausal age [46]. AMH is a product of the granulosa cells of the antral follicles, and serum AMH levels decline with age and not only reflect the number of early and developing antral follicles but also earlier stages of follicle development [47]. AMH concentration may be a novel marker of ovarian ageing [48]. However, our results indicated that a later menopausal age is associated with diabetes. These discrepant findings might be related to the age limit of our study. Our study investigated elderly women aged 60 years and older; therefore, we cannot be certain about the exact sequence of diabetes and menopause.

Our results suggest that there was an association between menopausal age and gastroenteritis. A study by Lichtarowocz et al. [49] showed that women with Crohn’s disease had an earlier menopausal age when compared with healthy women from the same area. This study indicates that early menopausal age is associated with chronic gastroenteritis.

Our research findings should be interpreted with an understanding of the limitations of recall bias and the nature of cross-sectional data. Self-reported information tends to be affected by recall bias, and those too ill to participate in the investigation were excluded. Responses for demographic and lifestyle factors obtained in cross-sectional studies may also be misclassified, as they can reflect the participants’ status at the time of the study; this may be an issue particularly in studies of elderly women compared with studies of women at or prior to menopause.

## 5. Conclusions

According to our results, birth year, education level, type of work, current smoking status, and BMI affected menopausal age. Being born in an earlier year, having a lower education, and engaging in physical labor were associated with an earlier menopausal age; however, a higher BMI was associated with later menopausal age. In addition, menopausal age was associated with diabetes and gastroenteritis in elderly women living in Northeast China.

## Figures and Tables

**Table 1:** Demographic and lifestyle factors, body mass index (BMI), and mental health in different menopausal age groups ^1^.

Variable	Total (*n*)	Menopausal Age						Statistics	
		≤45		46–53		≥54			
		*n*	%	*n*	%	*n*	%	χ^2^	*p*
Birth Year								23.696	0.008
1948–1952	1034	145	13.4	736	72.8	153	13.8		
1943–1947	579	108	20.2	390	67.3	81	12.5		
1938–1942	331	60	16.5	239	73.0	32	10.5		
1933–1937	155	38	25.4	99	63.2	18	11.4		
Married/cohabitation	1543	251	16.9	1075	70.0	217	13.0	1.080	0.663
Rural	1070	179	16.9	755	70.7	136	12.4	0.163	0.936
Education ≤ 9 years	1871	311	17.0	1321	71.2	239	11.8	10.276	0.015
Income								1.376	0.890
High	60	8	15.4	42	68.3	10	16.3		
Middle	895	153	17.7	634	70.3	108	12.1		
Low	1144	190	16.7	788	70.4	166	12.9		
Physical laborer	759	139	18.9	523	69.8	97	11.3	2.943	0.275
Current smoker	363	71	19.9	267	73.0	25	7.1	11.650	0.009
Current drinker	34	8	15.1	21	72.6	5	12.3	0.149	0.940
Irregular diet	191	38	19.6	130	70.2	23	10.2	1.523	0.455
Lack of physical exercise	756	122	16.6	536	71.5	98	11.9	0.864	0.733
BMI (kg/m^2^)								22.169	0.003
<25	1154	193	17.2	833	73.0	128	9.9		
25 to <30	787	131	17.5	534	67.8	122	14.8		
≥30	158	27	15.4	97	63.7	34	20.9		

^1^ Multiple Rao-Scott *χ*^2^ tests were used with the medium menopausal age as the reference. Numbers are unweighted, but percentages are weighted.

**Table 2:** Chronic diseases and mental health in different menopausal age groups ^1^.

Variable	Total (*n*)	Menopausal Age						Statistics	
		≤45 ^2^		46–53 ^2^		≥54 ^2^			
		*n*	%	*n*	%	*n*	%	*χ*^2^	*p*
Anemia	60	9	15.5	44	73.9	7	10.5	0.443	0.813
Diabetes	394	69	17.0	250	65.2	75	17.7	11.823	0.007
Hyperlipidemia	1019	178	18.4	703	69.2	138	12.5	2.115	0.448
Hypertension	1298	211	17.4	899	69.1	188	13.5	3.065	1.213
Ischemic heart disease	631	114	18.2	437	70.5	80	11.3	1.869	0.498
Cerebrovascular disease	363	65	18.8	256	70.0	42	11.2	1.215	0.594
COPD	193	39	24.7	138	67.5	16	7.8	10.973	0.067
CGPU	290	55	20.5	208	72.3	27	7.1	10.915	0.051
Cholecystitis/gallstone	370	65	16.9	261	72.3	44	10.8	1.351	0.574
Arthritis	434	87	20.4	295	68.8	52	10.9	4.531	0.154
Chronic low back pain	408	71	17.8	276	68.9	61	13.3	0.488	0.816
Poor mental health	644	122	19.5	448	69.7	74	10.8	8.634	0.033
Any disease ^3^	1972	328	17.2	1378	70.3	266	12.5	0.498	0.817

^1^ Multiple Rao-Scott *χ*^2^ tests were used with the medium menopausal age as the reference. ^2^ Numbers are unweighted, but percentages are weighted. ^3^ “Any disease” was defined as one or more of the 11 chronic diseases or poor mental health. COPD: chronic obstructive pulmonary disease; CGPU: chronic gastroenteritis/peptic ulcers.

**Table 3:** OR and 95% CI of socio-demographic and lifestyle factors in relation to menopausal age ^1^.

Variable	≤45 vs. 46–53 (Years)	≥54 vs. 46–53 (Years)
Birth Year		
1948–1952	1.00	1.00
1943–1947	1.708 (1.205, 2.420) *	0.943 (0.673, 1.321)
1938–1942	1.340 (0.922, 1.946)	0.794 (0.499, 1.264)
1933–1937	2.445 (1.525, 3.921) *	1.111 (0.620, 1.991)
Married/cohabitation	1.125 (0.815, 1.553)	1.151 (0.801, 1.652)
Rural	0.905 (0.646, 1.268)	1.051 (0.723, 1.529)
Education ≤ 9 years	0.729 (0.466, 1.139	0.515 (0.327, 0.812) *
Income		
Low	1.00	1.00
Middle	1.079 (0.774, 1.506)	0.769 (0.538, 1.099)
High	0.824 (0.353, 1.926)	0.806 (0.361, 1.800)
Physical laborer	1.413 (1.021, 1.957) *	0.898 (0.636, 1.269)
Current smoker	1.189 (0.848, 1.669)	0.577 (0.347, 0.959) *
Current moderate/heavier drinker	0.800 (0.280, 2.288)	1.283 (0.471, 3.498)
Irregular diet	1.179 (0.759, 1.831)	0.890 (0.539, 1.468)
Lacking physical exercise	0.921 (0.634, 1.338)	0.955 (0.683, 1.337)
BMI (kg/m^2^)		
<25	1.00	1.00
25 to <30	1.138 (0.845, 1.532)	1.565 (1.152, 2.125) *
≥30	1.099 (0.642, 1.882)	2.440 (1.482, 4.016) *

^1^ Complex weighted computation was used in the statistical analysis. * *p* < 0.05.

**Table 4:** OR and 95% CI of specific chronic diseases and mental health in relation to menopausal age adjusted for socio-demographic and lifestyle factors ^1^.

Chronic Disease	≤45 vs. 46–53 (Years)	≥54 vs. 46–53 (Years)
Anemia	0.893 (0.421, 1.895)	0.902 (0.396, 2.054)
Diabetes	1.072 (0.768, 1.495)	1.611 (1.142, 2.274) *
Hyperlipidemia	1.179 (0.897, 1.548)	0.953 (0.713, 1.274)
Hypertension	1.031 (0.778, 1.367)	1.164 (0.853, 1.588)
Ischemic heart disease	1.036 (0.765, 1.403)	0.818 (0.589, 1.136)
Cerebrovascular disease	1.139 (0.810, 1.602)	0.876 (0.586, 1.311)
COPD	1.608 (0.909, 2.846)	0.627 (0.349, 1.128)
CGPU	1.235 (0.764, 1.996)	0.533 (0.333, 0.855) *
Cholecystitis/gallstone	0.999 (0.695, 1.436)	0.753 (0.511, 1.109)
Arthritis	1.256 (0.908, 1.738)	0.808 (0.549, 1.188)
Chronic low back pain	1.100 (0.780, 1.552)	1.038 (0.732, 1.473)
Poor mental health	1.272 (0.953, 1.696)	0.898 (0.643, 1.255)
Any disease ^2^	1.116 (0.642, 1.938)	0.822 (0.427, 1.582)

^1^ Complex weighted computation was used in the statistical analysis. ^2^ “Any disease” was defined as one or more of the 11 chronic diseases or poor mental health. COPD: chronic obstructive pulmonary disease; CGPU: chronic gastroenteritis/peptic ulcers. * *p* < 0.05.
